# Regulation of Partial and Reversible Endothelial-to-Mesenchymal Transition in Angiogenesis

**DOI:** 10.3389/fcell.2021.702021

**Published:** 2021-10-07

**Authors:** Jennifer S. Fang, Nan W. Hultgren, Christopher C. W. Hughes

**Affiliations:** ^1^Department of Molecular Biology and Biochemistry, University of California, Irvine, Irvine, CA, United States; ^2^Department of Ophthalmology, Stein Eye Institute, David Geffen School of Medicine at University of California, Los Angeles, Los Angeles, CA, United States; ^3^Department of Biomedical Engineering, University of California, Irvine, Irvine, CA, United States

**Keywords:** EndoMT, EndMT, endothelial, mesenchymal, cell identity, cell plasticity, angiogenesis, partial EndoMT

## Abstract

During development and in several diseases, endothelial cells (EC) can undergo complete endothelial-to-mesenchymal transition (EndoMT or EndMT) to generate endothelial-derived mesenchymal cells. Emerging evidence suggests that ECs can also undergo a partial EndoMT to generate cells with intermediate endothelial- and mesenchymal-character. This partial EndoMT event is transient, reversible, and supports both developmental and pathological angiogenesis. Here, we discuss possible regulatory mechanisms that may control the EndoMT program to dictate whether cells undergo complete or partial mesenchymal transition, and we further consider how these pathways might be targeted therapeutically in cancer.

## Introduction

Endothelial cells (EC) that line the various blood vessels of the body share numerous structural characteristics and gene expression programs, yet are also remarkably heterogeneous (Aird, 2012; Kalucka et al., 2020; Paik et al., 2020) and plastic (Paruchuri et al., 2006; van Meeteren and ten Dijke, 2012; Dejana et al., 2017; Andueza et al., 2020). Blood vessel development is a multi-step process involving dynamic changes in EC morphology and gene expression to drive coalescence of primitive ECs into a primordial network (vasculogenesis), followed by EC proliferation and network expansion (angiogenesis). Lastly, blood vessels reorganize and mature into a hierarchal network architecture (remodeling and specification). During these processes as well as in the context of certain diseases, ECs undergo diversification, and some even take on a new non-EC identity (Welch-Reardon et al., 2015; Gritz and Hirschi, 2016; Piera-Velazquez and Jimenez, 2019; Qiu and Hirschi, 2019; Kenswil et al., 2021). In complete endothelial-to-mesenchymal transition (EndoMT), for example, activation of a central EndoMT program – similar to the program that drives epithelial-to-mesenchymal transition (EMT) in epithelial cells – induces a subpopulation of ECs to fully abandon their EC identity and transition to a mesenchymal cell phenotype (Welch-Reardon et al., 2015; Piera-Velazquez and Jimenez, 2019). This process generates several mesenchymal cell types (Tran et al., 2012; Yao et al., 2013) including endothelial-derived fibroblasts (Zeisberg et al., 2007b; Li et al., 2009; Hashimoto et al., 2010; Aisagbonhi et al., 2011; Moore-Morris et al., 2014; Chen et al., 2016) critical for embryonic tissue development (Timmerman et al., 2004) and disease progression in atherosclerosis (Souilhol et al., 2018), vascular fibrosis (Chen et al., 2012), and cancer (Zeisberg et al., 2007a).

In addition to supporting complete mesenchymal transition, the EndoMT program can also proceed only partially resulting in the temporary and reversible appearance of intermediate cells that exhibit both endothelial and mesenchymal characteristics (Tombor et al., 2021). Tombor et al. (2021), for example, recently used single-cell transcriptomic analysis to show that a subpopulation of ECs transiently adopt a mesenchymal signature within 7 days following myocardial infarction, but that they return to baseline EC identity by 14 days post-infarction rather than fully committing to a mesenchymal fate. While there are several possible explanations for the transient EndoMT signature in this study, one possibility is that partial EndoMT activation following myocardial infarction supports the robust injury-induced activation of acute new vessel growth (i.e., angiogenesis) typically observed in such models (Manavski et al., 2018; Li et al., 2019; Zou et al., 2019). Indeed, we and others have proposed, based on a growing body of evidence, that angiogenic EC undergo partial EndoMT during healthy and pathological angiogenesis to support new vessel formation (Welch-Reardon et al., 2014, 2015; Hultgren et al., 2020). Consistent with this hypothesis, both Manavski et al. (2018) and Li et al. (2019) recently observed in separate lineage-tracing studies that clonal expansion of ECs is enhanced in myocardial infarction-induced neovascularization. Using transcriptomic analysis, Manavski et al. (2018) further found enrichment of EndoMT genes in regions of clonally expanded vessels. By contrast, although Li et al. (2019) also observed enhancement of some mesenchymal genes in single-cell RNA-Seq study of post-infarction EC, they failed to note significant differences in overall EndoMT signature at their assessed timepoint, potentially due to the generally shallower sequencing depth of scRNAseq which may miss key transcription factors.

Activation of the EndoMT program in EC during angiogenesis must be partial and reversible to support the formation of perfused and functional blood vessels. This implies the existence of essential regulatory mechanisms that act on the EndoMT program to determine whether it will proceed completely in some tissues, but only partially in other contexts such as angiogenesis. Here, we revisit the argument for sprouting angiogenesis as a partial EndoMT event and explore possible regulatory mechanisms that might limit complete progression through the EndoMT program during angiogenesis, thereby preventing excessive mesenchymal transition and ensuring organized and controlled new vessel growth.

## Sprouting Angiogenesis as a Partial EndoMt Event

Sprouting angiogenesis is a complex developmental program wherein specialized endothelial “tip” cells migrate away from the parent vessel wall toward pro-angiogenic stimuli, while bringing with them endothelial “stalk” cells, thereby establishing a new blood vessel sprout (Gerhardt et al., 2003). Endothelial tip and stalk cells are dynamic and transient states (Jakobsson et al., 2010) determined by each cell’s relative Dll4-activated Notch signaling (Hellstrom et al., 2007; Suchting et al., 2007) and downstream VEGFR1/2 expression levels (Williams et al., 2006). Cells that successfully outcompete adjacent cells for the tip cell position do so through classical Notch-mediated lateral inhibition. Specifically, tip cells strongly express Dll4 to activate high levels of Notch signaling in their neighbors, which are thereby activated to take on the phenotype of stalk cells (Blanco and Gerhardt, 2013) allowing for sprout elongation.

Given the heterogeneous outcomes associated with EMT program activation, identification of an EMT event can be difficult and requires the combined observation of several cellular and molecular hallmarks, including the upregulated expression of at least one of several EMT-associated transcription factors, induced expression of mesenchymal markers, and alterations in functional properties such as cytoskeletal rearrangement, reduced cell-cell adhesions, and increased cell motility (Yang et al., 2020). Furthermore, cells with epithelial-mesenchymal plasticity – that is, with mixed epithelial and mesenchymal character indicating partial EMT – must in addition to the above hallmarks also retain cellular and molecular aspects of epithelial identity such as persistent expression of some epithelial markers and/or residual cell-cell junctions (Yang et al., 2020).

When compared to this standard, the cellular and molecular changes that occur in sprouting angiogenesis are highly suggestive of partial EndoMT (Potenta et al., 2008; Welch-Reardon et al., 2014, 2015; Piera-Velazquez and Jimenez, 2019; Hultgren et al., 2020). During angiogenesis, tip cells retain EC marker expression even while they undergo a dramatic loss of typical EC morphology and function. These changes include altered cell shape and polarity, extension of numerous filopodia that guide cell migration (Gerhardt et al., 2003), destabilization of cell-cell junctions (del Toro et al., 2010; Bentley et al., 2014; Hultgren et al., 2020) as well as increased cell motility (Jakobsson et al., 2010; Welch-Reardon et al., 2014) and upregulated expression of mesenchymal markers [e.g., Smooth Muscle α-actin, or αSMA (Li et al., 2009; Mendoza et al., 2016)] and extracellular matrix degradation proteases (del Toro et al., 2010; Welch-Reardon et al., 2014). Furthermore, tip cells and trailing stalk cells migrate as a train toward external VEGF, a process reminiscent of the collective migration that occurs when epithelial cells undergo EMT during organogenesis as well as in invasive tumors. In a sense, one end of a tip cell – specifically, the leading edge – acquires a mesenchymal-like phenotype, while the trailing edge retains some endothelial characteristics including maintenance of junctional contacts to trailing stalk EC.

In further support of angiogenesis as a partial EndoMT event, we have found that the master transcription factors Snail and Slug are induced in ECs during sprouting angiogenesis, both developmentally (Hultgren et al., 2020) and in the malformed vasculature of growing tumors (Parker et al., 2004; Lu et al., 2007). More recently, our group reported that Slug, although only transiently required for developmental angiogenesis, is critical for pathological angiogenesis, such that absence of Slug leads to a striking lack of tumor vasculature which profoundly limits tumor expansion (Hultgren et al., 2020). We further showed that EC Slug overexpression downregulates adhesion and cell-cell junction proteins, and upregulates pathways associated with cell cycle, cell shape changes, and motility (Hultgren et al., 2020). Other master transcription factors are likely also involved in partial EndoMT during angiogenesis. For example, Ma W. et al. (2020) recently identified PAK4-induced Zeb1 as a driver of mesenchymal gene expression in glioblastoma EC. Taken together, these recent data strongly support the hypothesis that during sprouting angiogenesis, an EndoMT program is partially and reversibly activated in angiogenic ECs to support acquisition of the subset of mesenchymal characteristics necessary to form new vessel sprouts. This model further implies that tight regulatory mechanisms must govern partial EndoMT events in normal angiogenesis to control the degree of mesenchymal transition, thereby maintaining an organized program of new vessel growth.

## Regulatory Mechanisms That Govern the EndoMt Program

### Signaling Pathways Regulating EndoMT

Similar to its role in EMT, TGF-β superfamily signaling is a potent activator of the EndoMT program, both in developmental and pathological settings (Arciniegas et al., 1992; van Meeteren and ten Dijke, 2012; Xiao et al., 2015; Pardali et al., 2017). This process is mediated by both canonical and non-canonical Smad-dependent downstream pathways (Medici et al., 2011; Piera-Velazquez and Jimenez, 2019) and necessary for organogenesis in many developmental contexts (Niessen et al., 2008; Piera-Velazquez and Jimenez, 2019). Other ligands, including Wnt (Liebner et al., 2004; Aisagbonhi et al., 2011), FGF (Correia et al., 2016; Paik et al., 2020), NFK B (Mahler et al., 2013; Cho et al., 2018), and ET-1 (Widyantoro et al., 2010) also regulate EndoMT, with many of these signals appearing to act in parallel with or converging upon TGF-β and BMP signaling (Arciniegas et al., 1992; Hopper et al., 2016) to drive full mesenchymal transition.

Pro-angiogenic signaling pathways modulate the EndoMT program in many ways. VEGF/VEGFR2 signaling is a potent pro-angiogenic signal critical for healthy vascular development, and is abnormally upregulated in tumor vasculature of many cancer types in association with aggressive blood vessel growth (Shibuya, 2013). VEGF/VEGFR2 signaling regulates numerous cellular functions in EC such as cell survival, proliferation, migration, and junctional integrity via a constellation of downstream signaling modules including PI3K/Akt, PLC-γ/PKC, p38MAPK, ERK, RAC, FAK, JNK, and RhoA pathways (Abhinand et al., 2016). TGF-β signaling can influence VEGF/VEGFR2 signaling directly via canonical activation of intracellular Smads that target several downstream genes, including VEGFR2 (Mandriota et al., 1996) and (in the presence of hypoxia) VEGF (Sanchez-Elsner et al., 2001). TGF-β can also indirectly affect VEGF/VEGFR2 signaling via non-canonical effects on many of the same modules activated by VEGF/VEGFR2, including PI3K/Akt, p38MAPK, ERK, and JNK (Ma J. et al., 2020). VEGF also exerts positive feedback on TGF-β1 expression via PI3K/Akt activation (Li et al., 2005). Taken together, these findings underscore the potential for complex crosstalk between VEGF and TGF-β signaling. We have previously described the interrelationship between the VEGF, Notch, and TGF-β pathways and how this lays the foundation for the sprouting phenotype (Holderfield and Hughes, 2008) and thereby the ensuing EndoMT program. In general, VEGF and TGF-β act synergistically (Holderfield and Hughes, 2008) and VEGF is required for TGF-β-induced angiogenesis *in vivo* (Ferrari et al., 2009). However, TGF-β can also antagonize VEGF/VEGFR2 signaling either directly by inhibiting VEGFR2 expression (Mandriota et al., 1996) or indirectly by antagonizing its cellular effects (Holderfield and Hughes, 2008). For example, VEGF is typically a pro-survival signal, but in the context of co-stimulation with TGF-β it becomes pro-apoptotic (Ferrari et al., 2006, 2009). This suggests that while VEGF plays a crucial role (including downstream of TGF-β) in initiating and driving angiogenesis, VEGF and TGF-β may also antagonize one another to determine the extent of EndoMT progression within the context of angiogenic EC. In support of this idea, exogenous VEGF treatment prevents TGF-β-induced EndoMT during cardiac fibrosis (Illigens et al., 2017) and VEGFR2 expression is reduced in glioblastoma vessels alongside EndoMT program activation and acquired mesenchymal marker expression (Liu et al., 2018).

In contrast to VEGF/VEGFR2, pro-angiogenic HIF-1α (Xu et al., 2015b) and TNFα (Sainson et al., 2008; Yoshimatsu et al., 2020) promote (rather than antagonize) complete EndoMT. Still other pro-angiogenic signals exert a context-dependent effect on the EndoMT program. HGF/c-Met signaling, for example, prevents TGF-β1-induced EndoMT in cardiac fibrosis (Okayama et al., 2012; Wang et al., 2018) but c-Met signaling activates EndoMT and abnormal vessel growth in glioblastoma (Huang et al., 2016) potentially compensating for the loss of VEGFR2 in these tumor vessels described by Liu et al. (2018). CXCL12 (SDF-1α)/CXCR4 signaling is upregulated in radiation-induced EndoMT in tumor vasculature where it is required for tumor-associated macrophage recruitment (Choi et al., 2018), and knockdown of either of CXCL12’s receptors, CXCR4 and CXCR7, disrupts angiogenic sprouting *in vitro* (Hultgren et al., 2020). Consistent with this, we found that CXCL12 activation of CXCR4 – but, interestingly, not CXCR7 – is required for CXCL12-induced upregulation of master transcription factor, Slug, which drives partial EndoMT during angiogenesis (Hultgren et al., 2020). By contrast, others have reported that CXCL12 activation of CXCR7 induces Wnt signaling to inhibit EndoMT-associated fibrosis (Shen et al., 2020).

### Master Transcription Factors of EndoMT

Perhaps the key determinant for EndoMT is not any specific receptor pathway activation, but the extent to which the combined and integrated signaling through these pathways converge at the level of the EndoMT master transcription factors. These include members of the Snail family of zinc-finger transcription factors, Slug and Snail, as well as zinc-finger transcription factors, Zeb1 and Zeb2, and the basic helix-loop-helix transcription factor, Twist1 (Piera-Velazquez and Jimenez, 2019). These transcription factors were originally described as potent drivers of EMT (Lamouille et al., 2014) with more recent studies finding that their expression in ECs induces EndoMT (Piera-Velazquez and Jimenez, 2019) suggesting a common mesenchymal transition program in both cell types.

In epithelial cells there is significant cross-talk between Slug, Snail, Zeb1, Zeb2, and Twist1 to regulate EMT progression (Peinado et al., 2007). In ECs, the interrelationship between these transcription factors is less well understood, but likely similarly complex (Weinstein et al., 2020). Some studies propose that Snail is the primary driver of the EndoMT program, with other transcription factors playing a largely ancillary role to Snail. Global (Carver et al., 2001), epiblast- (Lomeli et al., 2009) and endothelial-specific Snail (Wu et al., 2014) knockout is embryonic lethal due to profound defects in cardiovascular development (although this may or may not be due to an EndoMT defect), whereas animals lacking Slug survive embryogenesis with comparatively subtle defects in blood vessel development (Hultgren et al., 2020). In cultured ECs, TGF-β2 induces Snail expression via Smad-mediated MEK/ERK, PI3K, and p38MAPK (Medici et al., 2011) but does not significantly upregulate Slug and Twist1 (Kokudo et al., 2008). Snail is also strongly upregulated by hypoxia, and is directly targeted by HIF-1α during induction of EndoMT in corneal ECs (Xu et al., 2015b). Gene silencing approaches show that Snail is necessary for both TGF-β2- (Kokudo et al., 2008) and low shear stress-activated (Mahmoud et al., 2017) EndoMT. Furthermore, in several of these models, Slug expression is at least partially dependent on Snail (Xu et al., 2015b; Mahmoud et al., 2017) indicating cross-talk between Snail- and Slug-mediated EndoMT signaling.

Our studies as well as others, on the other hand, suggest that Slug and Snail both play important and non-redundant (if over-lapping) roles in EndoMT (Welch-Reardon et al., 2014; Hultgren et al., 2020; Weinstein et al., 2020). Slug and Snail negatively regulate one another’s expression (Chen and Gridley, 2013a,b) and both participate in distinct (as well as shared) signaling circuits that govern partial and full EndoMT (Weinstein et al., 2020). Further supporting the hypothesis that Slug and Snail function independently in EndoMT, endothelial Snail expression is unaffected by transgenic ablation of Slug in the retinal microvasculature (Hultgren et al., 2020) although it can compensate for Slug during heart formation in embryogenesis (Niessen et al., 2008). Instead, transcriptomic analysis indicates that Slug regulates a distinct suite of genes during early angiogenesis consistent with induction of partial EndoMT, including upregulation of mesenchymal markers (e.g., αSMA) as well as pro-proliferative and pro-migratory genes, and destabilization of endothelial junction genes (e.g., Occludin) without concurrent suppression of endothelial markers such as PECAM-1 (Hultgren et al., 2020).

Aside from Slug and Snail, endothelial expression of Zeb1 (Singh et al., 2019; Fu et al., 2020), Zeb2 (Chen et al., 2010), and Twist1 (Mahmoud et al., 2016) also promote EndoMT and angiogenesis. Endothelial expression of Twist1 induces a partial EndoMT program in ECs in response to TGF-β2 stimulation, resulting in increased EC proliferation and migration, a more mesenchymal-like cell morphology, and downregulation of endothelial junction proteins (Mammoto et al., 2018). Similarly, endothelial Twist1 overexpression drives mesenchymal marker expression in pulmonary ECs, and is necessary for the development of vascular structures in an implanted fibrin gel model of blood vessel network formation (Mammoto et al., 2020). Fu et al. (2020) also found that endothelial deletion of Zeb1 leads to improved vascular normalization and reduced cancer progression in various tumor models by reducing tumor vessel density and permeability, mainly by reducing TGF-β signaling in ECs and associated tumor stroma. More recently, Ma W. et al. (2020) found that PAK4 drives mesenchymal gene expression in the EC of glioblastoma blood vessels, and that in this setting, Zeb1 (but not Slug) is required for PAK4 suppression of cellular adhesion proteins leading to increased vascular permeability.

Experimental studies as well as *in silico* analysis suggest that Twist1 may operate upstream of Slug and Snail (Sanchez-Elsner et al., 2001; Okayama et al., 2012; Huang et al., 2016). For example, Mammoto et al. (2018) found that Twist1 upregulates Slug expression, which the authors propose is a necessary intermediate step for EndoMT to proceed in these cells. Yet, our transcriptomic analysis of Slug overexpressing ECs also indicate positive feedback by Slug onto Twist1 expression (Hultgren et al., 2020). By contrast, Zeb1 and Zeb2 appear to function primarily downstream of these transcription factors (Lee et al., 2018; Weinstein et al., 2020). In corneal ECs, Zeb1 is required for Snail upregulation of cell cycle and extracellular matrix proteins during EndoMT (Lee et al., 2018). Thus, while the sequential as well as lateral relationships between Snail transcription factors, Zeb transcription factors, and Twist1 suggest significant cross-talk, it currently remains unclear exactly how these signals coordinately regulate EndoMT progression. Further studies are necessary to fully elucidate the (clearly complex) relationship between their expression patterns and functions in EndoMT.

### Notch

Notch signaling appears to play a central – and still largely unclear – function during EndoMT. Signaling via Notch requires cell-cell contact between membrane-bound Notch ligands (e.g., Dll4 and Jag1) and cell surface Notch receptor expressed on adjacent cells. Thus, Notch signaling necessarily requires that at least some cell-cell junctions be intact, suggesting an initiating role in EMT and presumably an early role also in EndoMT. Indeed, Notch drives EndoMT in development (Niessen et al., 2008; Chang et al., 2011) and during disease (Noseda et al., 2004; Liu et al., 2014). Yet, we and others have also found that Notch limits sprouting angiogenesis (Hellstrom et al., 2007; Suchting et al., 2007) and that small molecule inhibition of Notch signaling exacerbates EndoMT (Chen et al., 2015; Hultgren et al., 2020). Specifically, in our hands, Notch signaling inhibition combined with Slug overexpression leads to complete fragmentation of sprouts during *in vitro* angiogenesis, indicative of cells undergoing more aggressive or complete mesenchymal transition (Hultgren et al., 2020).

The solution to this conundrum likely lies in the way we have traditionally approached Notch signaling, which has thus far been presumed to be an *instructive* signal. More consistent with its multiple and differing roles in numerous developmental programs, however, would be if Notch signaling instead plays a *permissive* role to open a “window of opportunity” for other pathways to be active. Thus, for any process – whether positively or negatively acting pathways – both could each be dependent on Notch signaling, such that neither is able to operate without a permissive signal from Notch. In EC, Notch may thus control whether pro- or anti-mesenchymal transition signals gain the upper hand. Similar processes have been suggested for Notch during arteriovenous specification (Fang et al., 2017) hemogenesis (Gama-Norton et al., 2015) and endocrine cell specification (Dutta et al., 2008).

### MicroRNA

MicroRNAs (miRNA) are short single-stranded, non-coding RNA sequences that regulate post-transcriptional gene expression at the mRNA level. Several miRNAs are involved in EMT (Nicoloso et al., 2009), both through regulation by – and feedback onto – Snail (Gill et al., 2011; Chen D. D. et al., 2019) Slug (Chen D. D. et al., 2019) and Zeb genes (Burk et al., 2008). Multiple miRNA species have recently been identified as positive and negative regulators of TGF-β-induced EndoMT (Kim, 2018; Glover et al., 2019) – including miR-630 which inhibits EndoMT by directly targeting Slug (Sun et al., 2016) – suggesting that the dynamic and integrated signal from multiple miRNAs acting in concert may also determine the extent of EndoMT and other EC fate changes. Furthermore, FGF signaling has been shown to promote TGF-β mediated EndoMT via regulation of let-7 miRNA expression (Chen et al., 2012) but to limit it via miR-20 (Correia et al., 2016). Consistent with this hypothesis, endothelial expression of Dicer – the protein responsible for miRNA pre-processing – is required for angiogenesis (Suárez et al., 2008) and miRNAs have also recently been reported to regulate endothelial-to-hematopoietic transition (Kasper et al., 2020). Thus, the interrelationship between miRNA species in ECs and their combined effect on EndoMT progression warrant further study.

### Epigenetic Modifications

Both complete and partial EndoMT depend upon the availability of key inducers to activate core DNA-binding transcription factors that regulate the expression of downstream mesenchymal transition genes. Given this, epigenetic changes that alter access to individual genes (or chromatin, more broadly) play a significant role at multiple levels of the EndoMT program, such as to regulate the availability of EndoMT inducers and effectors (Turunen and Yla-Herttuala, 2011; Lewandowski et al., 2015; Schwanbeck, 2015). For example, during normal heart development, HDAC3-mediated recruitment of EZH2 leads to transcriptional silencing of TGF-β1 to prevent aberrant EndoMT (Lewandowski et al., 2015). Other angiogenic signals that influence the EndoMT pathway – including VEGF and Notch pathway effectors – are also sensitive to DNA modifications (Turunen and Yla-Herttuala, 2011; Schwanbeck, 2015). Lastly, DNA methylation status can also regulate the levels of EMT-associated master transcription factors in epithelial cells (Lee and Kong, 2016) suggesting that similar regulation might occur in ECs to influence EndoMT.

Endothelial-to-mesenchymal transition progression is also sensitive to epigenetic changes that alter access to mesenchymal genes acted upon by EndoMT master transcription factors (Maleszewska et al., 2015). Under both developmental and pathological settings, TGF-β alters the methylation of EndoMT-related genes (Maleszewska et al., 2015; Xu et al., 2015a), and can (either alone or alongside Notch co-stimulation) induce histone acetylation (Fu et al., 2009). More fundamentally, the comparative difference in EndoMT transcription factor importance in physiological versus pathological angiogenesis (Hultgren et al., 2020; Ma W. et al., 2020) may be due to differences in chromatin architecture during development versus under inflammatory and other disease settings, such as in cancer. Alternatively, inflammation-induced epigenetic changes may also explain why the EndoMT program proceeds to completion in some disease contexts such as atherosclerosis or cancer, but not in angiogenesis. Indeed, the complexity and heterogeneity in cell type-specific responses to common inflammatory signals such as NFK B is established by the epigenetic landscape that uniquely determines enhancer region accessibility across distinct tissues (Natoli, 2009; Natoli et al., 2011). Epigenetic changes also occur during carcinogenesis to drive tumor growth and vascularization (Baylin and Jones, 2016). How chromatin remodeling under these and other pathological settings influence the accessibility of mesenchymal transition genes in EC, thereby altering the outcome of EndoMT program activation, remains unclear.

## Model of Partial EndoMt Regulation

Early studies of EMT and EndoMT proposed that mesenchymal transition is a binary, on/off switch between two distinct cell states. However, more sophisticated studies have now refuted that idea, and instead support an alternative model wherein most cell fate changes – including both EMT and EndoMT – involve progressive transition from one cell identity to another via a fluid spectrum of intermediate cell states (Lamouille et al., 2014; Sha et al., 2019). In support of this latter model, initiation of EMT in pluripotent epithelial stem cells through downregulation of the adherens junction protein E-cadherin triggers expression of Slug and related transcription factors, leading to the acquisition of some mesenchymal characteristics; yet, these cells still retain expression of epithelial stem cell markers indicating only a partial EMT (Aban et al., 2021).

**FIGURE 1:**
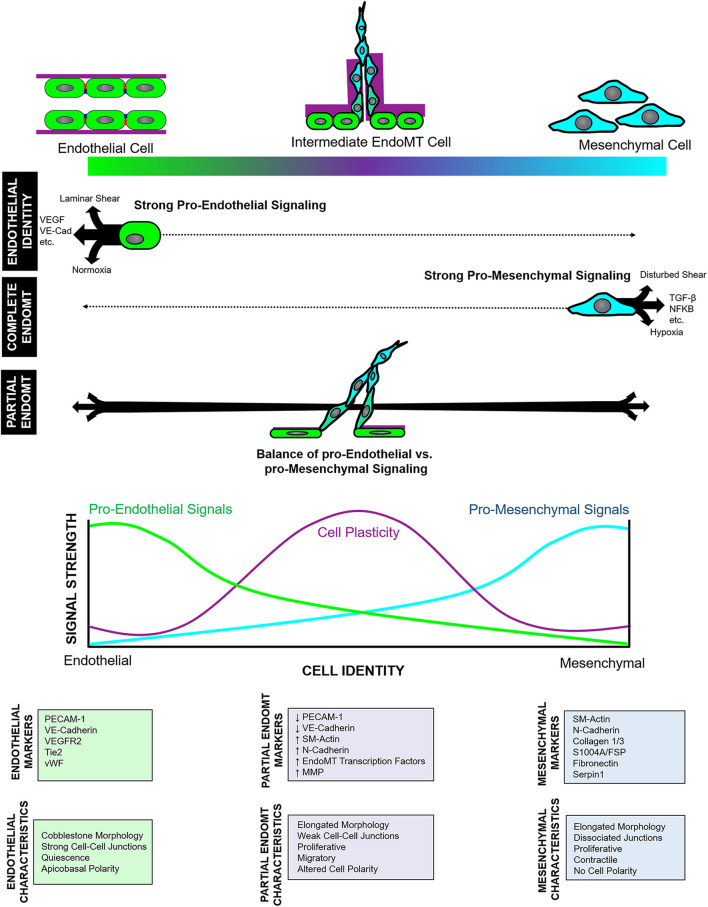
Model of endothelial-to-mesenchymal transition (EndoMT) regulation. EndoMT describes fluid transition between endothelial and mesenchymal identities and involving a spectrum of intermediate states wherein cells acquire a mixture of endothelial and mesenchymal character and marker expression. Complete vs. partial EndoMT is established by both the relative level of pro-endothelial and pro-mesenchymal signals that “push” or “pull” cells toward either endpoint of the endothelial-mesenchymal spectrum, as well as by the extent of cell plasticity.

Similarly, complete EndoMT also involves fluid transition through intermediate endothelial and mesenchymal substates with characteristics of both cell types. Cell subpopulations that co-express endothelial- and mesenchymal- markers – indicating cells in an intermediate state of EndoMT – have been reported in cardiac (Widyantoro et al., 2010), pulmonary (Mendoza et al., 2016), and dermal (Manetti et al., 2017) fibrosis, as well as in fetal valve endothelial progenitor cells (Paruchuri et al., 2006). Furthermore, pseudotime analysis of single cell transcriptomic and epigenomic data shows a spectrum of intermediate EndoMT cells, as well as ECs undergoing apparent transition to other non-mesenchymal cell types (Andueza et al., 2020; Kenswil et al., 2021). Given this, partial EndoMT is most likely not its own distinct process. Instead, we propose that partial EndoMT is best described as an incomplete activation and/or progression of the core EndoMT program, wherein regulatory signals are activated to limit the EndoMT process and prevent EC from fully transitioning into a mesenchymal endpoint. Clearly, in some contexts such as atherosclerosis and tumorigenesis, EndoMT program activation proceeds fully to completion. This may be due to chronic activation of the EndoMT program in this setting which (perhaps further augmented by the initiation of positive feedback mechanisms) drives cells rapidly through intermediate endothelial-mesenchymal states to promote robust transition into fibroblasts (Schwartz et al., 2018). Yet, in angiogenesis, we propose that the EndoMT program is only weakly or incompletely activated, or that regulatory “brakes” may inhibit EndoMT program progression to suppress complete mesenchymal transition. Together, this may ensure that cells proceed only partially through the endothelial-to-mesenchymal identity spectrum, enabling temporary and reversible adoption of a hybrid endothelial-mesenchymal cell state (Figure 1). To achieve such context-dependent nuance, the EndoMT program must be under strict regulatory control. We propose two layers of regulation on this process: (1) regulation of endothelial and mesenchymal identity signaling; and (2) regulation of cell plasticity to determine target cell sensitivity to those identity cues.

### Regulation of Endothelial and Mesenchymal Identity Signaling

Cell identity – even among committed cells – is dynamically established and maintained by the moment-to-moment balance of competing cell fate signals (Lee et al., 2014; Watanabe et al., 2014; Dejana et al., 2017). Indeed, ECs can respond rapidly to changes in local cell fate signals (Dejana et al., 2017) which suggests that EC identity maintenance remains a largely active process involving continuous input from local endothelial identity cues that include ligand-receptor signaling (i.e., VEGF, FGF, BMP, etc.), cell-matrix signaling, perivascular cell signaling, fluid shear stress, and microenvironmental oxygen content (Dejana et al., 2017). EC fate change programs (such as EndoMT) must overcome these signals to release cells from their EC commitment and redirect them toward other cell lineages. Thus, in the context of complete EndoMT, activation of the EndoMT program in ECs must generate mesenchymal fate cues with sufficient magnitude to outcompete endothelial identity signals and drive cells fully through the endothelial-to-mesenchymal identity spectrum to adopt a mesenchymal identity. By contrast, partial EndoMT occurs when the “push” and “pull” of endothelial and mesenchymal cues are momentarily balanced, thereby resulting in cells situated in an intermediate, uncommitted equilibrium state mid-way between endothelial and mesenchymal identities (Figure 1). Thus, we might imagine endothelial and mesenchymal identity signals functioning as two competing rheostats to establish target cell identity: fully “turning up” mesenchymal identity signals will successfully drive mesenchymal transition toward completion, especially if endothelial identity signals are simultaneously “turned down.” However, if mesenchymal identity signaling are only weakly “turned up” – as would occur if regulatory mechanisms limit or antagonize activation of the EndoMT program – cells would only undergo partial EndoMT.

Several EndoMT signaling pathways likely regulate EndoMT progression by modulating the relative strength of endothelial vs. mesenchymal fate signals. Classic EndoMT activators that drive complete EndoMT to generate endothelial-derived fibroblasts (such as TGF-β, hypoxia, and inflammatory signals, etc.) likely do so by strongly inducing pro-mesenchymal cues that overpower endothelial commitment signals, perhaps through the initiation of positive feedback loops. Others, however, may only modestly activate mesenchymal transition, either alone or in combination with the maintenance of endothelial identity signals, to limit the EndoMT program and ensure it proceeds only partially. VEGF, for example, is a classic pro-angiogenic factor that may promote (controlled and healthy) vessel growth by activating the EndoMT program to induce sprouting angiogenesis, while simultaneously preserving endothelial identity cues to prevent excessive mesenchymal transition in resulting tip and stalk cells. Underscoring its role in promoting new vessel growth, we found that VEGF signaling blocking prevents overaggressive sprouting in the context of Slug overexpression (Hultgren et al., 2020) suggesting that VEGF synergizes with Slug in driving EndoMT. Furthermore, VEGF is strongly overexpressed in cancer, where it is linked to appearance of the disorganized, aggressive hypersprouting typical of the tumor vasculature. Yet, in support of a secondary role for VEGF in securing endothelial identity in angiogenesis, some studies have found VEGF to antagonize TGF-β signaling in EndoMT (Paruchuri et al., 2006). For example, fetal valve progenitors are bipotential cells that co-express endothelial and mesenchymal markers suggesting an intermediate EndoMT state; this plasticity is lost in adult cells (Paruchuri et al., 2006). Using these cells, Paruchuri et al. (2006) found that VEGF promotes (reversible) transition toward greater EC identity, in part by antagonizing TGF-β-induced mesenchymal transition. VEGF similarly limits TGF-β-mediated fibrosis in cardiac tissue (Illigens et al., 2017). Taken together, these data suggest that the distinction between controlled vs. uncontrolled angiogenesis is determined by the precise level of local VEGF signaling such that the EndoMT program is activated only partially, and in a limited and regulated way.

Another possible regulatory mechanism that might prevent complete EndoMT may involve the relative levels of master transcription factors, Slug and Snail. Slug and Snail negatively regulate one another’s expression (Chen and Gridley, 2013a,b), and both participate in distinct (as well as shared) signaling circuits that govern partial and full EndoMT (Weinstein et al., 2020). Using an *in vitro* sprouting angiogenesis assay, we found that Slug upregulation precedes that of Snail by several days, suggesting that Slug activation is independent of Snail. Moreover, Slug dominates during the early proliferative phase of angiogenesis, whereas in later stages of angiogenesis, its expression decreases as Snail expression increases (Welch-Reardon et al., 2014). Consistent with this finding, Snail upregulation is also delayed following TGF-β activation of complete EndoMT in cultured ECs (Sobierajska et al., 2020). Thus, the dynamic balance of Slug vs. Snail levels appears to modulate the EndoMT response to dictate the extent of (partial vs. complete) mesenchymal transition. Whereas physiologic levels of Slug support early sprouting angiogenesis via induction of a partial EndoMT gene signature, high and prolonged overexpression of Slug – particularly in combination with Notch signaling inhibition – drives full dissociation of ECs from angiogenic sprouts, suggesting that Slug levels tightly regulate the extent of EC response to EndoMT signals (Hultgren et al., 2020). Meanwhile, delayed Snail upregulation during angiogenesis (Welch-Reardon et al., 2014) and in response to exogenous TGF-β-mediated EndoMT activation (Sobierajska et al., 2020) suggests that Snail functions later than Slug, perhaps to support a more robust EndoMT activation signal that pushes cells more aggressively down the EndoMT pathway. Consistent with this possibility, Snail has a higher affinity for target DNA binding sites than does Slug, suggesting that when expressed, Snail may exert a more potent effect to transactivate mesenchymal genes (Bolos et al., 2003).

### Regulation of Cell Plasticity

The extent of EndoMT progression likely also depends upon cell plasticity, which acts as a permissive signal to determine the extent to which cells are sensitive to any sort of fate change signal (Figure 1). Although EC identity is mostly actively maintained, EC commitment is also controlled in progenitor cells by epigenetic modification to more stably alter accessibility of endothelial lineage genes (Ohtani et al., 2011). Highly committed EC may thus be relatively insensitive to cell identity transition cues. By contrast, cell plasticity signals that “loosen” cell commitment will enhance cell stemness, which may create a necessary window of opportunity that enables EndoMT signals to act upon the target cell. In support of this idea, transcriptomic analysis finds that cells in intermediate EMT states are more stem-like than cells on either end of the epithelial or mesenchymal identity spectrum (Jolly et al., 2014). In other words, cells in partial mesenchymal transition (whether by EMT or EndoMT) may be situated at a cell fate inflection point, wherein they are less committed and therefore especially responsive to external identity cues that might push them either fully toward distinct mesenchymal programs or reverse the transition back toward their original cell identity.

This model may further explain why ECs are heterogeneous in their response to EndoMT program activation, despite being exposed to the same EndoMT activation signal. For example, Xiao et al. (2015) found that TGF-β-induced EndoMT yields subpopulations of both SMA-expressing myofibroblasts as well as SMA-negative fibroblasts, suggesting that EndoMT intermediate states differ in their responsiveness to mesenchymal identity cues. Further, Pinto et al. (2018) recently reported that the extent of EndoMT induction via TGF-β/Snail overexpression differs according to the tissue-specific identity of cultured primary ECs. EC commitment may differ according to EC tissue origin, which may underlie their distinct responsiveness to EndoMT activation. In other words, while some ECs exposed to EndoMT cues may transition fully toward a mesenchymal fate, others in the same population may be more resistant to EndoMT activation and may transition only partially through the EndoMT program, pause in an intermediate EndoMT state, or even reverse course and return to an EC identity.

Although the signaling pathways that regulate cell plasticity in EndoMT remain unidentified, one possibility is that Notch signaling serves this function to prime, or permit, ECs to respond to EndoMT cues (and other cell fate signals) by creating a window of opportunity, as suggested above (Dejana et al., 2017). Notch is critically involved in a context- and tissue-dependent manner throughout embryonic development, including for its well-established role in maintaining stem and progenitor cell pluripotency to regulate the outcome of cell fate decisions (Koch et al., 2013). In ECs, Notch signaling is associated with acquisition and/or maintenance of arteriovenous (Fang et al., 2017; Fang and Hirschi, 2019), lymphatic (Murtomaki et al., 2014), and hemogenic (Gama-Norton et al., 2015; Gritz and Hirschi, 2016) identity, and is also required for EndoMT (Timmerman et al., 2004). The outcome of Notch signaling in ECs is also ligand-dependent: Dll4 and Jagged activation of Notch have opposing roles on angiogenesis, where Dll4-Notch signaling induces EC quiescence but Jagged-Notch signaling is both pro-angiogenic and pro-proliferative (Benedito et al., 2009). As mentioned, high levels of Dll4-Notch signaling by tip cells laterally inhibits adjacent stalk cells, preventing them from similarly adopting the mesenchymal-like tip cell phenotype. More recently, *in silico* modeling suggests that cancer cells undergoing EMT are associated with high Jagged-Notch signaling levels that establish a “window of stemness” wherein hybrid epithelial-mesenchymal cells transiently adopt a more stem-like state (Bocci et al., 2018). Thus, in the context of angiogenesis, Dll4-Notch may normally preserve EC identity, and suppression of this signal through direct repression of Dll4 by Slug (Hultgren et al., 2020) may loosen EC commitment to permit cells to respond to mesenchymal transition cues, thereby enabling EndoMT to (at least partially) proceed. Consistent with this possibility, *in silico* modeling finds that perturbations that downregulate Dll4 are associated with partial EndoMT in modeled ECs (Weinstein et al., 2020). Subsequent regulation of Notch activation levels (perhaps via Jagged) may then determine whether cells remain in an intermediate stage of EndoMT, reverse course back to a committed EC identity, or transition fully toward a mesenchymal state, either by exerting further effects on cell plasticity alone and/or through cross-talk with TGF-β, VEGF or other EndoMT regulatory pathways (Holderfield and Hughes, 2008). Further studies are necessary to test these possibilities.

## Dysregulated EndoMt in Pathological Angiogenesis

Mature blood vessels are highly stable, and sprouting angiogenesis is a tightly regulated process that involves the temporary adoption by angiogenic ECs of pro-proliferative and migratory states (via stringent regulation of the EndoMT program). This enables the formation of new vessel sprouts, but also the return of EC to a quiescent and stable state (Schwartz et al., 2018). However, in hypervascular diseases such as diabetic retinopathy and cancer, ECs are persistently destabilized and developmental angiogenic pathways (including the EndoMT program) are reactivated in an aberrant and dysregulated fashion, leading to unrestrained and pathological growth of abnormal and disorganized blood vessels (Schwartz et al., 2018). This suggests that pathological and hyperaggressive angiogenesis may represent a loss of regulatory control over the EndoMT program. In cancer, for example, uncontrolled EndoMT may result in angiogenic ECs that acquire excessive mesenchymal character, leading to aggressive, uncontrolled, and disorganized sprouting to produce the highly aberrant and leaky vasculature characteristic of tumors.

Consistent with this hypothesis, we recently reported that beyond its developmental role in angiogenesis, Slug is critically required to support the pathological hypersprouting of blood vessels into tumor explants. In the absence of endothelial Slug expression, tumor angiogenesis was almost completely abolished (Hultgren et al., 2020) underscoring the importance of Slug-mediated partial EndoMT signaling to enable sprouting angiogenesis in cancer. Indeed, both our group and others have found that individual EndoMT transcription factors may be largely dispensable during development, but still play a central and indispensable role in the context of pathological angiogenesis, suggesting that regulatory mechanisms that ordinarily compensate for their loss are no longer intact (Hultgren et al., 2020; Ma W. et al., 2020). For example key developmental genes often have multiple (shadow) promoters driven by different transcription factors. Epigenetic shut-down of one of these promoters may then render expression of the gene susceptible to loss of a previously redundant transcription factor. Thus, therapies designed to limit pathological angiogenesis in a variety of diseases may be more effective if they also target Slug, Snail, or other master EndoMT transcription factors or their downstream signaling pathways.

## Therapeutic Potential of Targeting EndoMt Regulatory Mechanisms for Pathological Angiogenesis

Endothelial-to-mesenchymal transition is associated with several disease settings, including atherosclerosis, where activation of the EndoMT program drives robust EC transformation into EC-derived fibroblasts that are critical for plaque formation and progression (Souilhol et al., 2018). Studies of mouse atherosclerotic models have shown that EC in atheroprone regions are induced to express EndoMT transcription factors (Mahmoud et al., 2016, 2017) suggesting that EndoMT is a significant contributor to the fibroblast population in this setting. Consistent with this, Chen et al. (2012) found, somewhat surprisingly, that the TGF-β pathway is required for EC inflammation and atherosclerotic plaque development. Furthermore, they found that genetic knockout or silencing-RNA knockdown of this pathway reduced inflammation, and both prevented and rescued atherosclerotic plaque formation, making the EndoMT pathway, and specifically the TGF-β component, an attractive target for therapeutic treatment of this disease (Chen et al., 2012). Similarly, several drugs with anti-EndoMT properties are already approved for treatment of other diseases such as pulmonary fibrosis (Tsutsumi et al., 2019) and diabetes (Yao et al., 2018) and are further being considered as candidates for the treatment of cancer (Choi et al., 2020). Thus, anti-EndoMT approaches may also be effective in controlling pathological angiogenesis, including in cancer where angiogenic invasion into tumor masses drives further cancer growth and metastasis. However, since several clinically approved anti-EndoMT therapies are aimed at (and have been studied in the context of) preventing complete EndoMT – that is, the appearance of EC-derived fibroblasts to prevent fibrosis – it is unclear how effective existing treatment strategies will be in the context of pathological angiogenesis in which even a partial EndoMT program is sufficient to drive pathology. It is possible that additional, new strategies may be required to halt or reverse this process, potentially by acting early in the cascade of transcriptional events.

Existing anti-angiogenic approaches in cancer treatment have largely focused on inhibiting pro-angiogenic signals. When pathological angiogenesis is reconceptualized as a problem of dysregulated EndoMT – and not merely an issue of over-active pro-angiogenic (e.g., VEGF) signaling – it becomes clearer why anti-VEGF therapy [which has been a standard of care in cancer treatment for the last two decades (Welti et al., 2013)] largely fails to provide long-term control of tumor angiogenesis and cancer progression (Ferrara, 2010). Simply blocking VEGF signaling is not enough since the hypoxic and pro-inflammatory tumor microenvironment as well as the heightened mutation rate of cancer cells allows for rapid adaptation to anti-VEGF treatment through the activation of alternative pro-EndoMT “escape pathways” that rescue pathological angiogenesis in a VEGF-independent manner. Attempting to target and inhibit each pro-mesenchymal pathway individually would result in an inefficient game of “whack-a-mole.”

Most pro-EndoMT pathways appear to converge at the level of the master EndoMT transcription factors. Indeed, as mentioned above, several EndoMT transcription factors are abnormally expressed in tumor-associated ECs where they drive abnormal tumor vessel formation. Therefore, therapies that target master EndoMT transcription factors are likely to be more effective than treatments that inhibit EndoMT program activators, particularly in the context of cancer, because of the following properties of these transcription factors: (1) they serve as a common signaling nexus for most upstream pro-EndoMT activators and could therefore alleviate the issue of “escape pathway” activation; (2) they are transcription factors that directly drive expression of suites of cell identity genes, thus ensuring broad effects on target cells; and (3) their abnormal activation is often specific to the tumor environment (Welch-Reardon et al., 2014) which suggests that healthy vessels would be relatively protected from off-target effects.

One concern in this approach might be the challenge of targeting intracellular proteins. However, recent developments in antibody-mimicry and antibody-fusion peptides, as well as advances in viral- and nanoparticle-based delivery methods, have shown great promise for cell-type specific delivery of antibodies that target intracellular antigens (Slastnikova et al., 2018) and should allow for drug delivery specifically to tumor blood vessels that express unique markers relative to healthy vasculature. Recent advances in delivery of RNA-based therapeutic molecules also offer new possibilities for acute, temporary, and efficient knockdown of intracellular protein expression (Nature Medicine, 2019; Dammes and Peer, 2020). This latter approach may be especially feasible when targeting transcription factors like Slug, which have a short half-life and are only transiently expressed. Nonetheless, approaches that target Slug, Snail, Zeb1, Zeb2, and Twist1 – individually or collectively – to limit pathological angiogenesis must consider the likely complex (and still largely unclear) interrelationship between these transcription factors during angiogenesis. More work is therefore needed to better elucidate the cross-talk between master EndoMT transcription factors to enable more precise control of their function as an emerging therapeutic strategy.

Another possible avenue for therapeutic intervention in the EndoMT program may involve targeting regulatory mechanisms that govern EC plasticity. Therapies that selectively promote EC commitment and limit sensitivity to local pro-EndoMT signals may prevent overaggressive and pathological angiogenesis in the tumor mass, particularly when applied in combination with other chemotherapeutic or anti-angiogenic treatments. Such an approach might be used to either normalize tumor vessels, or to block tumor angiogenesis altogether. If, as we propose, Dll4-Notch signaling promotes EC commitment to limit (partial or complete) EndoMT, therapies that activate Notch signaling specifically in tumor vasculature may help suppress pathological angiogenesis. Yet, application of Notch inhibitors have produced mixed outcomes on tumor blood vessels (Bridges et al., 2011) likely due to the fact that Notch’s effect on cell plasticity appears to be both ligand- and context-dependent (Benedito et al., 2009). Currently available small molecule Notch inhibitors are overly broad in their suppression of all Notch activation. Blocking antibodies to the Dll4 or Jagged1 Notch ligand are more suitable for specific Notch signaling inhibition, although outcomes have thus far been surprising. Dll4 blockade, for example, promotes excessive tumor vessel growth consistent with a role for Dll4-Notch signaling in promoting EC commitment. However, vessels that result from Dll4-Notch inhibition are excessively disorganized and poorly perfused, which (somewhat unexpectedly) reduces tumor size (Bridges et al., 2011). Thus, further studies are needed to determine precisely what kind of Notch inhibition might be most effective to limit pathological angiogenesis in tumors; or, alternatively, whether targeting other pathways that regulate EC plasticity might be more suitable. Finally, to reiterate our earlier discussion on Notch signaling, activation or inhibition of Notch must always be considered in the context of what other signaling pathways may be active in the local environment that Notch signaling is now permitting or blocking.

## Discussion

Complete EndoMT serves as a critical source of endothelial-derived mesenchymal cells during organogenesis and is a significant contributor to fibrosis in disease. It is also increasingly clear that partial EndoMT also plays an essential function during angiogenesis (and likely other processes), and that dysregulation of the EndoMT program may contribute to abnormal and pathological blood vessel growth. Specifically, we propose that tight regulation of a core EndoMT program dictates the extent of mesenchymal transition in a context-appropriate manner by manipulating the strength of identity and transition cues, as well as the extent of target cell plasticity, and that loss of this control contributes to blood vessel disorganization in diseases such as cancer.

However, several questions remain outstanding: What are the contexts aside from angiogenesis under which partial EndoMT occurs? What specific regulatory mechanisms determine the extent of EndoMT progression, and how are they dynamically regulated during angiogenesis to maintain partial endothelial identity during sprouting? What are the regulatory mechanisms that govern cell plasticity during EndoMT, and how do they modulate progression of the EndoMT program? How stable and discrete are intermediate stages of partial EndoMT and, in addition to mesenchymal transition signals, are these cells sensitive to other cell lineage signals? Why do EndoMT transcription factors appear to be more essential in pathological angiogenesis compared to during development, and what aspects of the tumor microenvironment and other disease states lead to dysregulation of EndoMT regulatory mechanisms? And finally, how can EndoMT be targeted therapeutically in these diseases? An improved understanding of EndoMT, and specifically the regulatory mechanisms that dictate complete vs. partial mesenchymal transition in a context-dependent manner, warrant further study and are likely to reveal important insights into these and other crucial questions.

## Author Contributions

JF and NH wrote the manuscript. CH provided edits and additional scientific insights. All authors contributed to the article and approved the submitted version.

## Conflict of Interest

The authors declare that the research was conducted in the absence of any commercial or financial relationships that could be construed as a potential conflict of interest.

## Publisher’s Note

All claims expressed in this article are solely those of the authors and do not necessarily represent those of their affiliated organizations, or those of the publisher, the editors and the reviewers. Any product that may be evaluated in this article, or claim that may be made by its manufacturer, is not guaranteed or endorsed by the publisher.
